# Gender differences in the prevalence of congenital heart disease in Down’s syndrome: a brief meta-analysis

**DOI:** 10.1186/s12881-017-0475-7

**Published:** 2017-10-06

**Authors:** Tereza Cristina Pinheiro Diogenes, Felipe Alves Mourato, José Luiz de Lima Filho, Sandra da Silva Mattos

**Affiliations:** 1 Círculo do Coração de Pernambuco, Recife, Pernambuco Brazil; 2 Universidade Federal de Pernambuco (UFPE), Recife, Pernambuco Brazil; 3 Unidade de Cardiologia Materno e Fetal (UCMF), Av. Governador Agamenon Magalhães, 4760, Paissandu, PE CEP 52010-902 Brazil

**Keywords:** Down, Gender, Meta-analysis, Systematic review, Congenital heart disease

## Abstract

**Background:**

Down’s syndrome (DS) affects one per 700 live births and congenital heart disease (CHD) occurs in 40–60% of these patients. Contributing factors to the association between DS and CHD are being unraveled. Gender could be one of them.

**Methods:**

We performed a meta-analysis of CHD prevalence in DS, separated by gender. Three search engines were used and 578 articles were reviewed. Twelve articles were included.

**Results:**

Quantitative analysis showed a higher prevalence of CHD, particularly atrioventricular septal defects (AVSD), in female patients. No differences were found in others forms of CHD.

**Conclusion:**

CHD, particularly AVSD, are more common in the female gender of Down’s syndrome patients.

**Electronic supplementary material:**

The online version of this article (10.1186/s12881-017-0475-7) contains supplementary material, which is available to authorized users.

## Background

Down’s Syndrome (DS) affects one in each 700 live births [1, 2]. Its incidence is directly related to maternal age and has been increasing throughout the world [3]. Congenital Heart Disease (CHD) occurs in 40–60% of DS cases [4] and constitutes an important prognostic factor in these patients. Numerous factors may contribute to the development of different cardiac malformations in DS. Some are being unraveled recently in animal models [5]. However, until now, there has been few reports looking into the association of gender with CHD and DS.

It is well known that DS is a risk factor for CHD. In these patients, the most frequent forms of CHD are atrioventricular septal defects (AVSD), ventricular septal defects (VSD) and atrial septal defects (ASD) [6, 7]. The reported prevalence of these defects varies among studies [8–14]. This could reflect inherent characteristics of the studied populations, such a higher frequency of genetic variances that predispose to the presence of AVSD [5, 15–19].

In this context, gender could influence the presence and type of CHD. Some studies point out a higher predominance of male gender in patients with DS [20, 21], but studies carried out in pediatric cardiology centers point to a large number of female patients with DS and CHD [20, 22]. This paradox could be explained by a higher incidence of CHD in female patients with DS, leading to higher mortality rates earlier in life, although many other unknown factors could be at play to influence these findings.

The purpose of this study was to compare the prevalence of CHD and DS between genders through a meta-analysis and systematic review.

## Methods

### Eligibility criteria

Studies that described the prevalence of CHD in DS by gender were included. Studies where this information was not available were excluded.

### Information sources

The search for articles was performed using the following data engines: Medline (accessed via Pubmed), Scopus and Scielo. Terms included were those used by Mesh for Medline and Scopus, and the descriptors of Health Sciences (Decs) for Scielo. Terms included: “prevalence”, “Down syndrome” and “congenital heart disease”. Additional file 1 contains the full search strategy. Articles, published until August 30th, 2016, were included. Additional search was performed in the bibliographic references of the researched articles. Authors from selected papers with incomplete data were contacted by e-mail. Complete articles were obtained and analyzed by authors.

### Selection of studies and data extraction

Two authors (Mattos and Mourato) evaluated the title and abstracts of the identified articles. The complete texts of the selected abstracts were obtained and posteriorly analyzed by the same authors. After this initial analysis, each selected article’s information were added to a database. The authors agreed that discordances about the inclusion of an article should be sorted by consensus. However, there were no disagreements. Duplicated studies were excluded.

### Data analysis

Gender prevalence was calculated by dividing the total number of DS patients with CHD by the total number of DS for each gender. The prevalence of AVSD, ASD, VSD, PDA (patent ductus arteriosus) and TOF (tetralogy of Fallot) were calculated dividing the number of DS with each cardiac defect by the total number of patients with DS in the study. A combined data analysis was performed to identify the Odds Ratio between genders, being the female gender considered a risk factor. The confidence intervals and the size of the pondered effect were calculated and the meta-analysis graphs built using the MedCalc v 16.8 software.

Heterogeneity between studies was calculated using the I^2^, which describes the variability, not related to sample errors, in the studied population. An I^2^ beyond 75% is consistent with high heterogeneity. As such, the meta-analysis should be carried out using the fixed model if the heterogeneity analysis resulted in number constantly inferior to 75% and using a random effects model if it resulted in a number equal or superior to 75% (i.e. considering the I^2^ confidence interval). The Mantel-Haenszel method was used for calculating the weighted summary Odds ratio under the fixed effects model. Subsequently, heterogeneity statistics were incorporated to calculate the summary Odds ratio under the random effects model (in accordance to I^2^ statistics).

## Results

**Fig. 1:**
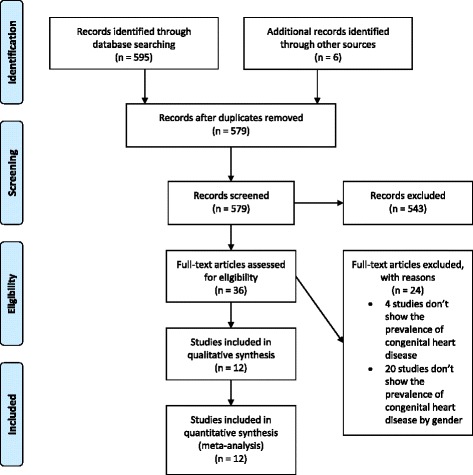
Flow chart of eligible studies for meta-analysis

From the engine database sources, 595 abstracts were selected. Initial review identified 35 for full text analysis. From this latter group, only four fulfilled the eligibility criteria and were included in the meta-analysis [22–25]. Direct contact with the authors of the remaining 31 articles made it possible to include another eight studies [12, 13, 20, 26–30], totalizing 12 articles for analysis. In total, 20,465 patients with DS (11,165 male and 9300 female) were included in the meta-analysis. Figure 1 demonstrates articles selection’s process and progress, according to the PRISMA method. Raw data were included in the Additional file 2.

**Fig. 2:**
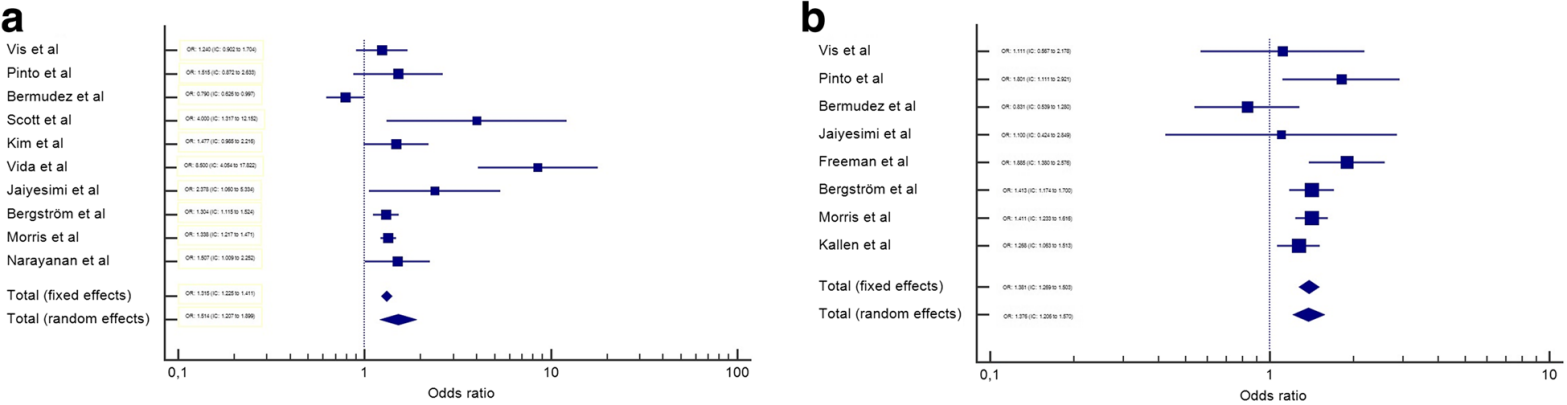
Meta-analysis of all CHD and AVSD by gender in Down syndrome. IC-Interval of confidence. OR-Odds Ratio. **a** – OR meta-analysis of all CHD in Down syndrome by gender. **b** – OR meta-analysis of AVSD in Down syndrome by gender

**Fig. 3:**
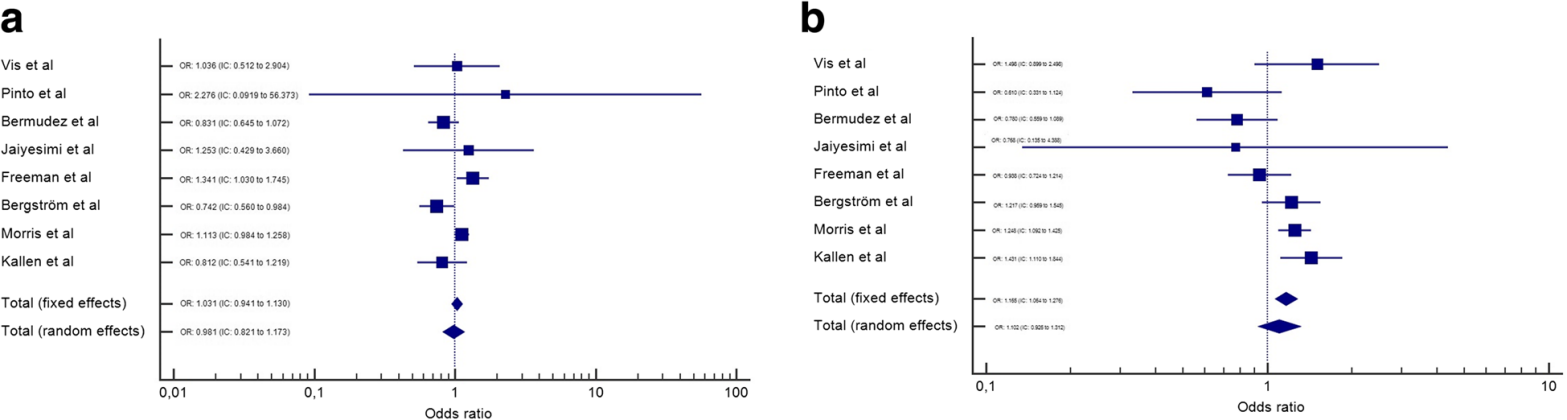
Meta-analysis of ASD and VSD by gender in Down syndrome. IC-Interval of confidence. OR-Odds Ratio. **a** – OR meta-analysis of ASD in Down syndrome by gender. **b** – OR meta-analysis of VSD in Down syndrome by gender

**Fig. 4:**
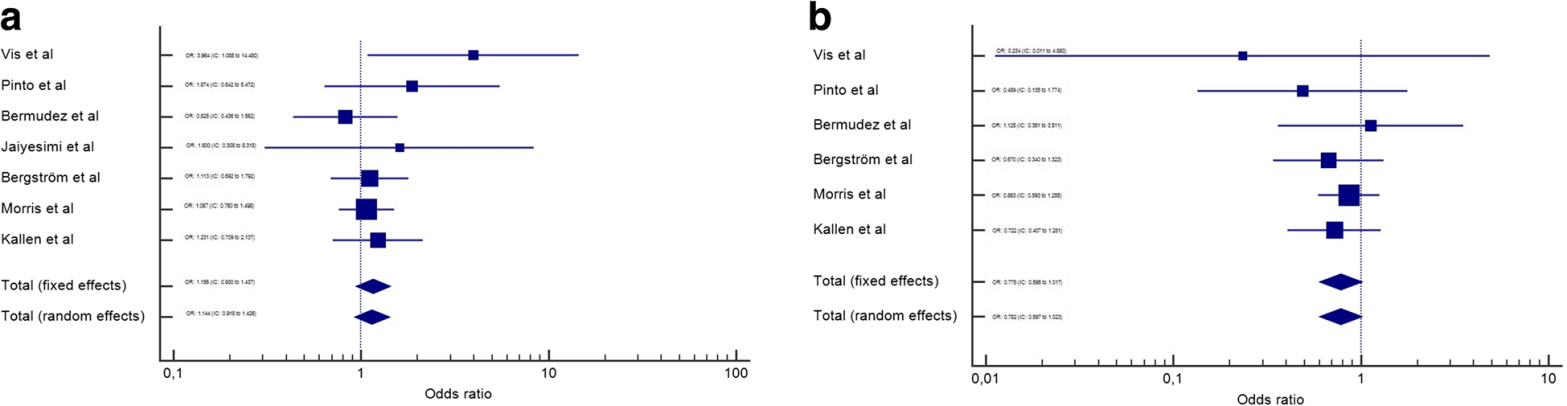
Meta-analysis of PDA and TOF by gender in Down syndrome. IC-Interval of confidence. OR-Odds Ratio. **a** – OR meta-analysis of PDA in Down syndrome by gender. **b** – OR meta-analysis of TOF in Down syndrome by gender

After the meta-analysis, it was observed that female gender is a risk factor for the presence of CHD in DS (Fig. 2). The same occurs if we consider AVSD alone (Fig. 2). However, when VSD, ASD, PDA and TOF (Figs. 3 and 4) are considered separately, there is no difference among genders. All analyses utilized the random effects’ model.

## Discussion

The frequency of CHD in this systematic analysis is in accordance with other studies involving DS [8, 10–12, 14, 20, 24, 28, 31–35]. Few studies fulfilled the inclusion criteria for this meta-analysis. The main problem was the lack of information about the prevalence of CHD in DS, according to gender. Some studies mentioned an association between the female gender and a higher prevalence of CHD [20, 22]. However, these findings were not highlighted subsequently.

Various theories exist to explain the origins of CHD in DS. Some authors suggest that the presence of certain variants in specific genes could be the underlying cause for CHD in this population [5, 36]. Others suggest a correlation with the presence of single nucleotides polymorphism (SNPs) and Copy Number Variations (CNVs) [18]. And there are also ethnical genetic differences, which could play a role in the different incidence of CHD among these patients [20, 24]. In this context, differences between gender, with their specific genetic charges, could also exert an influence over the determining factors for CHD in this population.

In this meta-analysis, we observed a higher frequency of CHD in the female gender (OR: 1.514, IC: 1.207 to 1.899). This finding suggests that this gender is more susceptible to CHD in DS. Another finding that supports this conclusion is that AVSD, alone, also showed a higher frequency in female gender (OR: 1.376, IC: 1.206 to 1.570); and this form of CHD is more prevalent in DS than the general population [37]. TOF, on the other hand, was more frequent in males, but without statistical significance (OR: 0.782, IC: 0.597 to 1.023). These differences might be explained by a potential different susceptibility of gender to different CHD pathogenic pathways (for example, AVSD is correlated with extracellular matrix anomalies and TOF with ectomesenquimal tissue migration anomalies [38]). Other possible explanation would be that males with DS die before birth or before the timing of these studies (with similar incidence early in life, but a lower prevalence later). Unfortunately, these hypotheses cannot be evaluated in this meta-analysis.

This meta-analysis had some limitations. First, it included only articles from three databases (Scielo, Pubmed and Scopus). This can lead to no identification of minor or locally published studies, whose inclusion could alter some of the findings presented herein (such as TOF being equally prevalent among genders in DS). Second, the populations included in this paper are rather different among themselves. For example, Morris et al. included data from 20 European Countries, and of both live births and abortions after the 20th week [25]. It is known that the prevalence of CHD, particularly complex malformations, is higher in abortions [39]. Conversely, Pinto et al. and Jaiyesimi et al. included children followed in health centers, which can overestimate the prevalence of CHD [22, 24]. Vis et al. included only adults with DS, which can reduce the prevalence of complex heart diseases (due to higher mortality) [23]. Third, it was not possible to perform an analysis of ethnicity separated by gender, which could provide more insights on the origin of CHD in DS. Despite such limitations, however, it was possible to show a clear trend of a higher prevalence of CHD (more specifically of AVSD) in the female population with DS.

## Conclusion

This brief meta-analysis demonstrated higher prevalence of congenital heart disease, particularly AVSD, on female patients with Down syndrome.

## Additional files

Additional file 1: Search strategy in Pubmed. Contains the terms and strategy to find the main articles of this meta-analysis in Pubmed. (DOC 51 kb)

Additional file 2: Raw data. Contains the raw data obtained from the articles for congenital heart disease and Down syndrome prevalence by gender. (DOCX 11 kb)

## Abbreviations

ASD: Atrial septal defects
AVSD: Atrioventricular septal defects
CHD: Congenital heart disease
CNV: Copy number variations
Decs: Descriptors of Health Sciences
DS: Down’s syndrome
OR: Odds ratio
PDA: Patent ductus arteriosus
SNPs: Single-nucleotide polymorphism
TOF: Tetralogy of fallot
VSD: Ventricular septal defects
