# Supplementary material for: Gender differences in the prevalence of congenital heart disease in Down’s syndrome: a brief meta-analysis
Source: BMC Med Genet. 2017 Oct 6;18:111. doi: 10.1186/s12881-017-0475-7 (PMC6389118; doi:10.1186/s12881-017-0475-7)
Supplement: Supplementary file 1 — Search strategy in Pubmed. Contains the terms and strategy to find the main articles of this meta-analysis in Pubmed. (DOC 51 kb) [file 12881_2017_475_MOESM1_ESM.doc]

Supplementary file 2: raw data

| Article | Author | Year | Male Down | Female Down | Male Down with CHD | Female Down with CHD | Male Down with ASD | Female Down with ASD | Male Down with VSD | Female Down with VSD | Male Down with AVSD | Female Down with AVSD | Male Down with PDA | Female Down with PDA | Male Down with TOF | Female Down with TOF |
| --- | --- | --- | --- | --- | --- | --- | --- | --- | --- | --- | --- | --- | --- | --- | --- | --- |
| Congenital heart defects are under-recognised in adult patients with Down’s syndrome | Vis et al | 2010 | 625 | 533 | 89 | 91 | 17 | 15 | 28 | 35 | 18 | 17 | 3 | 10 | 2 | 0 |
| Down’s syndrome: different distribution of congenital heart diseases between the sexes | Pinto et al | 1990 | 119 | 158 | 85 | 125 | 0 | 1 | 27 | 24 | 46 | 84 | 5 | 12 | 6 | 4 |
| Down syndrome: Prevalence and distribution of congenital heart disease in Brazil | Bermudez et al | 2015 | 607 | 540 | 338 | 269 | 192 | 150 | 96 | 69 | 52 | 39 | 23 | 17 | 6 | 6 |
| Incidence of Cardiac Lesions in Children 696 with Down’s Syndrome | Scott et al | 2014 | 28 | 35 | 14 | 28 | * | * | * | * | * | * | * | * | * | * |
| Prevalence of Congenital Heart Defects Associated with Down Syndrome in Korea | Kim et al | 2014 | 222 | 172 | 117 | 107 | * | * | * | * | * | * | * | * | * | * |
| Congenital cardiac disease in children with Down's syndrome in Guatemala | Vida et al | 2005 | 120 | 129 | 70 | 119 | * | * | * | * | * | * | * | * | * | * |
| Cardiovascular malformations in Omani Arab children with Down’s syndrome | Jaiyesimi et al | 2007 | 67 | 43 | 33 | 30 | 9 | 7 | 4 | 2 | 13 | 9 | 3 | 3 | * | * |
| Ethnicity, sex, and the incidence of congenital heart | Freeman et al | 2008 | 787 | 682 | * | * | 130 | 143 | 158 | 130 | 75 | 113 | * | * | * | * |
| Trends in Congenital Heart Defects in Infants With Down Syndrome | Bergström et al | 2016 | 1435 | 1153 | 727 | 660 | 139 | 85 | 157 | 150 | 284 | 298 | 37 | 33 | 24 | 13 |
| Major Congenital Anomalies in Babies Born With Down Syndrome: A EUROCAT Population-Based Registry Study | Morris et al | 2014 | 3905 | 3120 | 1579 | 1485 | 665 | 580 | 518 | 500 | 471 | 506 | 74 | 63 | 68 | 47 |
| Cardiac Spectrum, Cytogenetic Analysis and Thyroid Profile of 418 Children with Down Syndrome from South India: A Cross-sectional Study | Narayanan et al | 2013 | 235 | 183 | 134 | 122 | * | * | * | * | * | * | * | * | * | * |
| Major Congenital Malformations in Down Syndrome | Kallen et al | 1996 | 3015 | 2552 | * | * | 58 | 40 | 115 | 137 | 268 | 281 | 25 | 26 | 31 | 19 |
| Total | * | * | 11165 | 9300 | 3186 | 3036 | 1210 | 1021 | 1103 | 1047 | 1227 | 1347 | 170 | 164 | 137 | 89 |

Legend: *- Unavailable data. CHD- Congenital heart disease. ASD – atrial septal defect. VSD – Ventricular Septal Defect. AVSD – Atrioventricular septal defect. PDA- patent ductus arteriosus. TOF – Tetralogy of Fallot.
