# Supplementary material for: Gender differences in the prevalence of congenital heart disease in Down’s syndrome: a brief meta-analysis
Source: BMC Med Genet. 2017 Oct 6;18:111. doi: 10.1186/s12881-017-0475-7 (PMC6389118; doi:10.1186/s12881-017-0475-7)
Supplement: Supplementary file 2 — Raw data. Contains the raw data obtained from the articles for congenital heart disease and Down syndrome prevalence by gender. (DOCX 11 kb) [file 12881_2017_475_MOESM2_ESM.docx]

Table 1: Search strategy (Pubmed)

| (((((((((((epidemiology[Title/Abstract]) OR cohort study[Title/Abstract]) OR cohort analysis[Title/Abstract]) OR cross sectional study[Title/Abstract]) OR cross sectional analysis[Title/Abstract]) OR observational analysis[Title/Abstract]) OR prevalence[Title/Abstract]) OR frequency[Title/Abstract]))) AND ((((down syndrome[Title/Abstract]) OR mongolism[Title/Abstract]) OR trisomy 21[Title/Abstract]) OR down's syndrome[Title/Abstract])) AND ((((((congenital heart defects[Title/Abstract]) OR malformation of heart[Title/Abstract]) OR heart abnormalities[Title/Abstract]) OR congenital heart disease[Title/Abstract]) OR heart abnormality[Title/Abstract]) OR congenital heart defect[Title/Abstract]) |
| --- |
